# Identification of QTL for Grain Traits and Plant Height Using the Recombinant Inbred Line Population Derived from the Cross of Zhongke 331 × Nongda 399

**DOI:** 10.3390/ijms26083526

**Published:** 2025-04-09

**Authors:** Yi Liu, Yongxing Chen, Yijun Yang, Dan Qiu, Huaizhi Zhang, Jinghuang Hu, Guanghao Guo, Keyu Zhu, Hongkui Fu, Hongjie Li, Zhiyong Liu, Ruihui Wang, Qiuhong Wu

**Affiliations:** 1 College of Agronomy, Hebei Agricultural University, Baoding 071000, China; 18903196121@163.com; 2 Institute of Genetics and Developmental Biology, Chinese Academy of Sciences, Beijing 100101, China; yjy19972025@163.com (Y.Y.); qiudan@genetics.ac.cn (D.Q.); hzzhang@genetics.ac.cn (H.Z.); ghguo@genetics.ac.cn (G.G.); zhukeyu@genetics.ac.cn (K.Z.); hongkuifu@genetics.ac.cn (H.F.); zyliu@genetics.ac.cn (Z.L.); 3 Xianghu Laboratory, Institute of Biotechnology, Hangzhou 311231, China; chenyongxing@xhlab.ac.cn (Y.C.); hujinghuang@xhlab.ac.cn (J.H.); lihongjie@xhlab.ac.cn (H.L.); 4 College of Advanced Agricultural Sciences, University of Chinese Academy of Sciences, Beijing 100049, China

**Keywords:** *Triticum aestivum*, SNP, QTL, grain trait, plant height

## Abstract

Improving wheat yield is essential to meet the increasing demand for food production. This study aims to identify quantitative trait loci (QTL) associated with grain traits and plant height (PH) in winter wheat, using a recombinant inbred line (RIL) population derived from a cross between Zhongke 331 and Nongda 399. The RIL population was genotyped using the 16K GenoBaits Wheat single nucleotide polymorphism (SNP) array. A genetic linkage map was established, comprising 14,868 SNPs and spanning 3846.91 cM, with an average interval of 1.11 cM between markers. These SNPs were categorized into 3463 SNP bin markers, with 1653, 1508, and 302 located in the A, B, and D sub-genomes, respectively. QTL analysis for thousand-grain weight (TGW), grain length (GL), grain width (GW), and PH revealed 61 QTL influencing these traits across six environments. Loci *qPH-4B.1* and *qPH-4D.1* were consistently detected in five environments. QTL clusters with pleiotropic effects that regulate multiple grain traits were identified on chromosomes 4B and 4D. Furthermore, the combination of *qPH-4B.1* and *qPH-4D.1* resulted in a reduced plant height compared to the presence of either locus alone, indicating an additive effect between these loci.

## 1. Introduction

Wheat (*Triticum aestivum* L.) is crucial for global food security, providing over 20% of the world’s dietary calories [1]. With the global population expected to surpass 9 billion by 2050, wheat production must increase by 60% to meet rising food and nutritional demands [2]. China, which produces approximately 17% of the global wheat supply, faces unique challenges due to population growth and urbanization, which drive increased domestic demand [3,4]. Over the last two decades, the global food system has been increasingly strained by population growth and the reduction in arable land [5,6]. Therefore, breeding programs aimed at increasing wheat productivity and resilience are more important than ever.

Wheat yield, a complex trait influenced by multiple genes and their interactions with environmental factors, is a key priority for breeders [7]. Major components influencing yield include ears per unit area, grains per ear, and grain weight. Thousand-grain weight (TGW), a key yield determinant, is closely associated with grain length (GL) and grain width (GW), which are directly influenced by grain development [8,9,10,11]. Understanding the genetic mechanisms underlying these traits is essential for developing wheat varieties that combine high yield and improved grain quality.

Grain length is determined early in grain development and is relatively stable under varying environmental conditions, while GW and TGW, established later, are more influenced by environmental factors [12,13]. Grain size increases through changes in GW and GL, with adjustments in grain shape primarily derived from changes in GL [8]. Thousand-grain weight, a key yield determinant, also significantly impacts milling quality, processing performance, and seedling vigor [14,15]. Mapping quantitative trait loci (QTL) and identifying candidate genes that regulate these traits offers a promising approach to improving wheat yield and related characteristics.

The analysis of QTL is valuable for understanding complex quantitative traits [16]. Several studies have identified QTL for grain weight on various chromosomes, including a major QTL on chromosome 4B in different cultivars [17]. Gene *TaGS-D1* on chromosome 7DS regulates both GL and GW, leading to the improvement of grain size and yield [18]. The major QTL *Qtgw-cb.5A* on chromosome 5A increases grain weight by promoting the elongation of grain epidermal cells [19]. These discoveries demonstrate the effectiveness of QTL mapping in advancing wheat breeding programs aimed at improving yield-related traits.

Plant height (PH), which is closely associated with yield, was significantly reduced during the “Green Revolution” of the 1960s, leading to higher crop productivity. Twenty-six dwarf or semi-dwarf genes have been identified in wheat [20,21,22]. Among these, *Rht1* (*Rht*-*B1b*), *Rht2* (*Rht*-*D1b*), and *Rht8* are the most widely used dwarfing genes globally. While *Rht1* and *Rht2* reduce grain number per spike (GNS) and TGW, *Rht8* does not have these negative effects [23]. These results emphasize the value of QTL analysis in uncovering the genetic basis of grain yield and yield-related traits.

This study aims to identify QTL associated with grain-related traits and PH in a recombinant inbred line (RIL) population derived from two winter wheat cultivars, Zhongke 331 (ZK331) and Nongda 399 (ND399). By integrating a high-density SNP array with comprehensive phenotypic data collected across multiple years and environments, we intend to precisely map genetic loci governing these traits. The results are expected to provide valuable insights into the genetic basis of key yield-related traits and contribute to wheat improvement.

## 2. Results

### 2.1. Phenotypic Evaluation of the RIL Population

**Figure 1:**
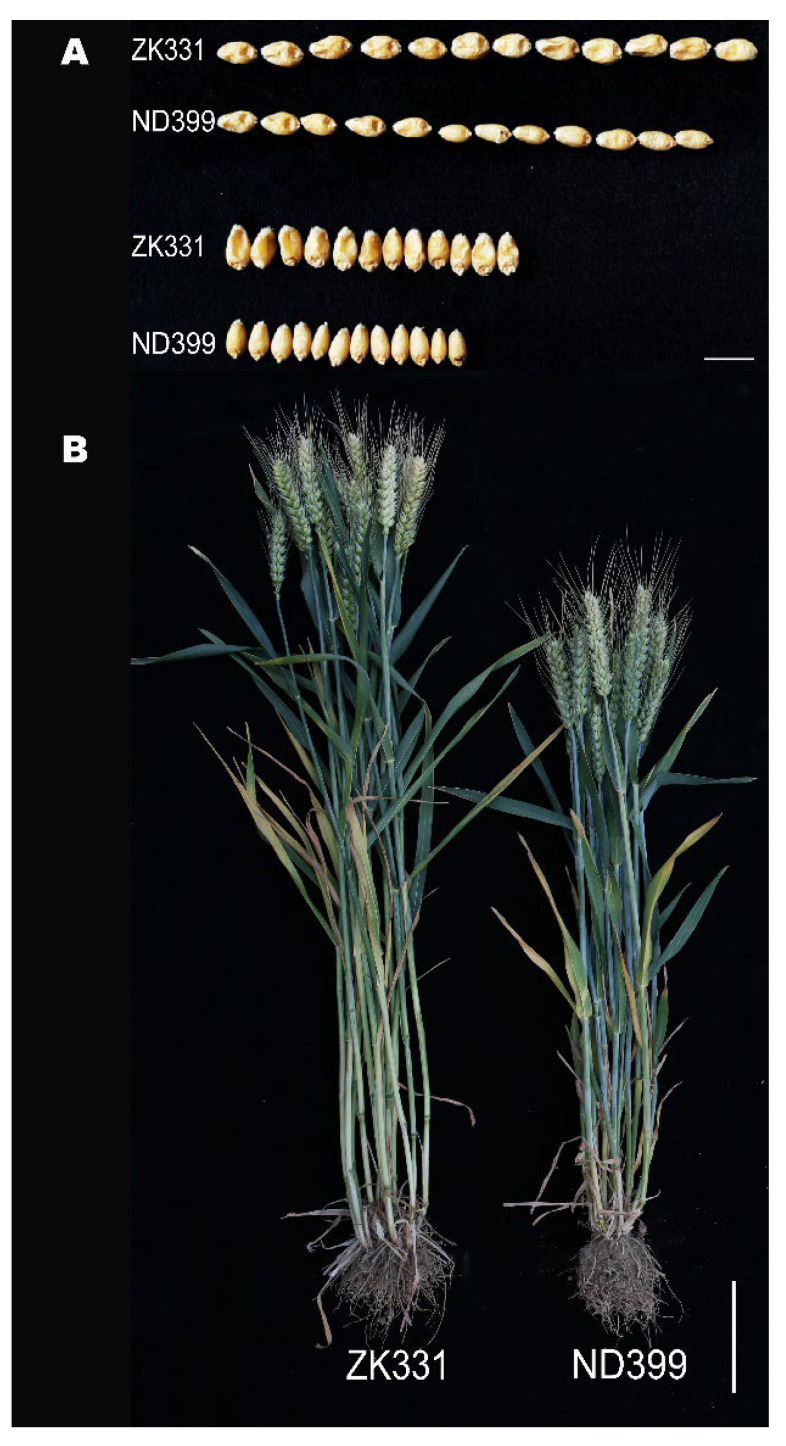
Comparison of grain length (GL) and grain width (GW) (**A**), and plant height (PH) (**B**), between Zhongke 331 and Nongda 399. The scale bar for (**A**) represents 1 cm, and the scale bar for (**B**) represents 10 cm.

**Figure 2:**
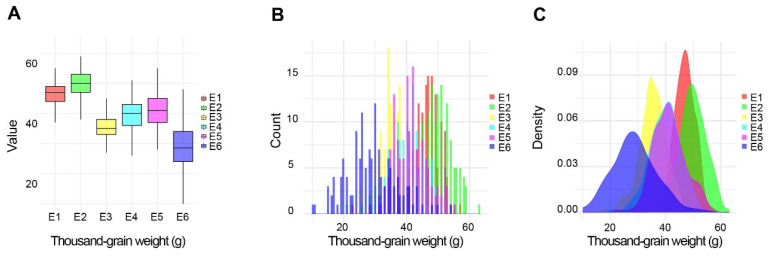
Histograms of the frequency distributions of grain traits and PH in the Zhongke 311 × Nongda 399 RIL population. TGW in the ZK331 × ND399 RIL population across different environments (**A**); frequency distributions of TGW in the RIL population under varying environmental conditions (**B**,**C**). E1 and E2: Shijiazhuang, Hebei Province (2020 and 2021); E3: Qingshui, Gansu Province (2021); E4: Anyang, Henan Province (2021); E5: Chengdu, Sichuan Province (2021); and E6: Hangzhou, Zhejiang Province (2021).

There were significant differences in TGW, GL, and GW in the ZK331 × ND399 RIL population across six environments, and significant differences in PH across five environments (*p* < 0.05). ZK331 consistently had higher values for these traits compared to ND399 under varying environmental conditions (Figure 1, Table 1). The broad-sense heritability for these traits, ranging from 0.76 to 0.93, indicated a strong genetic influence (Table 1). The RIL population also showed substantial genetic variation for all traits (Figure 2A and Figure S1). The frequency distributions of each trait were approximately normal across all environments, suggesting the nature of quantitative inheritance for these traits (Figure 2B,C, and Figure S1). Analysis of best linear unbiased estimators (BLUEs) revealed skewness and kurtosis values close to zero, supporting the hypothesis that these traits are controlled by multiple genetic loci.

### 2.2. Correlation Between Grain Traits and Plant Height

The correlations among TGW, GL, GW, and PH were evaluated across multiple environments (Table S1). Significant correlations were detected for these traits in most environments, with the strongest correlation between GW and TGW in environment E6 (*r* = 0.915, *p* < 0.01). TGW also had a strong correlation with GL. However, correlation coefficients varied across environmental conditions. For instance, the correlation between GL and PH was significant in only two out of six environments, indicating no significant relationship in the other conditions.

### 2.3. Construction of the Genetic Linkage Map

ZK331, ND399, and the RIL population were genotyped using the wheat 16K GenoBaits SNP array to create a high-density genetic map covering the entire wheat genome. The genetic map comprised 14,868 markers, which were subsequently reduced to 3463 markers across 21 chromosomes using the “BIN” function in IciMapping v4.2. The final genetic map spanned 3846.39 cM in length, with an average density of 3.09 cM/locus. The markers were distributed across the A, B, and D genomes, with 1653, 1508, and 302 markers covering genetic lengths of 1462.29, 1301.60, and 1083.02 cM, respectively. The largest number of localization markers was on chromosome 3B (537 markers), and the smallest was on chromosome 4D (33 markers). The longest covered chromosome was 3A (268.05 cM), and the shortest was 1D (74.75 cM) (Table S2, Figure S2).

### 2.4. QTL Mapping

We identified QTL for TGW, GW, and GL across five to six environments over two years using composite interval mapping. A total of 61 QTL controlling these traits were mapped to 19 wheat chromosomes, explaining 2.13% to 40.51% of the phenotypic variation (Table S3). Among them, 23 QTL were consistently detected across two or more environments, predominantly on chromosomes 1A, 1B, 2B, 2D, 3A, 3B, 3D, 4B, 4D, 5A, and 7A (Table 2, Figure S3). These stable QTL indicate key genomic regions that influence trait expression under diverse environmental conditions.

#### 2.4.1. Thousand-Grain Weight (TGW)

A total of 18 QTL for TGW were identified across chromosomes 1B, 2B, 3A, 3B, 4B, 4D, 5D, and 6A (Table S3). Five of these QTL, *qTGW-1B.1*, *qTGW-2B.1*, *qTGW-3B.1*, *qTGW-4B.1*, and *qTGW-4D.1* were consistently detected in two environments, indicating their stability. These stable QTL explained 7.93% to 18.65% of the phenotypic variation, with LOD scores ranging from 2.64 to 5.94. The additive effect of *qTGW-4D.1* was contributed by ND399, while the remaining four stable QTL had additive effects contributed by ZK331.

#### 2.4.2. Grain Length (GL)

For GL, we identified 19 QTL on chromosomes 1B, 2B, 2D, 3A, 3B, 4A, 5B, 6D, and 7A. Eight of these QTL, *qGL-1B.1*, *qGL-2B.1*, *qGL-2B.2*, *qGL-2D.1*, *qGL-3A.1*, *qGL-3B.1*, *qGL-3B.2*, *qGL-4A.1*, and *qGL-7A.1* were consistently detected across more than one environment (Table S3). These QTL accounted for 7.74% to 24.27% of the phenotypic variation, with LOD scores ranging from 2.67 to 19.92. The additive effects of *qGL-2B.2* were primarily contributed by ND399, while the other seven QTL effects were derived from ZK331.

#### 2.4.3. Grain Width (GW)

Fifteen QTL for GW were identified on chromosomes 1A, 1B, 2A, 2B, 2D, 3B, 3D, 4B, 4D, 5A, 7A, and 7D. Three of these QTL, *qGW-3D.1*, *qGW-4B.1*, and *qGW-4D.1*, were consistently detected across multiple environments, explaining 8.45% to 13.76% of the phenotypic variation, with LOD scores ranging from 2.75 to 4.07 (Table S3). The additive effect of *qGW-3D.1* and *qGW-4B.1* was contributed by ZK331, while *qGW-4D.1* had an additive effect contributed by ND399.

#### 2.4.4. Plant Height (PH)

We identified nine QTL for PH on chromosomes 1A, 3D, 4B, 4D, 5A, 7A, and 7B. Six QTL, *qPH-1A.1*, *qPH-3D.1*, *qPH-4B.1*, *qPH-4D.1*, *qPH-5A.1*, and *qPH-7A.1*, were consistently detected across multiple environments. These QTL explained 3.94% to 40.51% of the phenotypic variation (LOD = 2.60–16.42). *qPH-4B.1* and *qPH-4D.1* were detected in five environments with *qPH-4D.1* explaining the highest phenotypic variation (40.51%) (Table S3). The additive effects of QTL on chromosome 4D were derived from ND399, while the remaining five QTL originated from ZK331.

### 2.5. Co-Localization of QTL Controlling Different Traits

**Figure 3:**
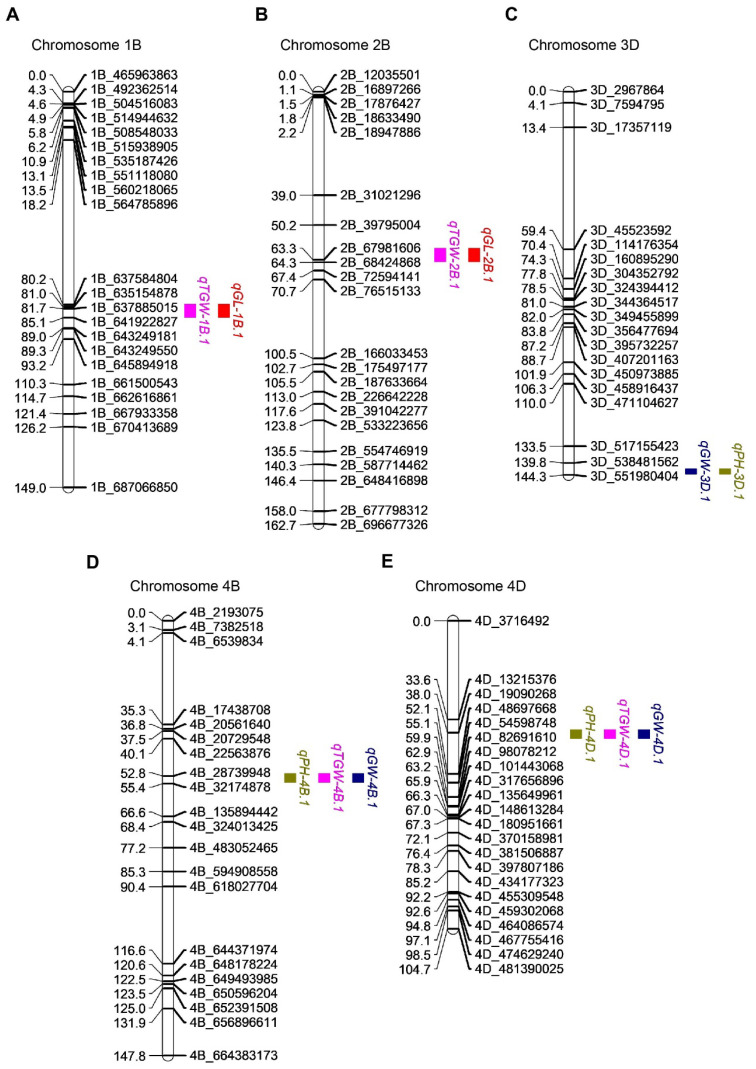
Co-localization of quantitative trait loci (QTL) associated with grain traits and PH. Co-localization of *qTGW-1B.1* and *qGL-1B.1* (**A**), co-localization of *qTGW-2B.1* and *qGL-2B.1* (**B**), co-localization of *qGW-3D.1* and *qPH-3D.1* (**C**), co-localization of *qTGW-4B.1*, *qGW-4B.1*, and *qPH-4B.1* (**D**), and co-localization of *qTGW-4D.1*, *qGW-4D.1*, and *qPH-4D.1* (**E**). TGW, GL, GW, and PH are marked in pink, red, dark blue, and green, respectively.

We found several QTL located within the same chromosome intervals, suggesting potential pleiotropy. For instance, *qTGW-1B.1* and *qGL-1B.1* were co-localized within the interval of 80–85 cM on chromosome 1B, explaining 11.38% to 24.27% of the phenotypic variation (LOD = 3.65–8.54). The additive effect controlling TGW and GL in this region was derived from ZK331 (Figure 3A). Similarly, *qTGW-2B.1* and *qGL-2B.1* were co-located on chromosome 2B (59–64 cM), explaining 8.69% to 14.63% of the phenotypic variation (LOD = 3.49–5.01) (Figure 3B). The additive effect on chromosome 2B controlling TGW and GL was mainly from ZK331. *qPH-3D.1* and *qGW-3D.1* were co-located on chromosome 3D (142–144 cM), explaining 9.29% to 15.70% of the phenotypic variation (LOD = 3.19–5.41) (Figure 3C). The additive effect on chromosome 3D controlling GW and PH was mainly from ZK331. Additionally, *qTGW-4B.1*, *qGW-4B.1*, and *qPH-4B.1* were mapped to the same interval (47–55 cM) on chromosome 4B, explaining 8.45% to 26.24% of the phenotypic variation with LOD scores between 2.75 and 15.81. The additive effect on plant height and TGW, GW, and PH on chromosome 4B was mainly from ZK331. On chromosome 4D, *qTGW-4D.1*, *qGW-4D.1*, and *qPH-4D.1* were mapped to the region of 36–45 cM, explaining 7.93–40.51% of the phenotypic variation (LOD = 2.64–16.42) (Figure 3D,E). The additive effect of the locus on chromosome 4D, which controls PH, TGW, and GW, was primarily from ND399.

### 2.6. Analysis of Additive Effects of the Major QTL

**Figure 4:**
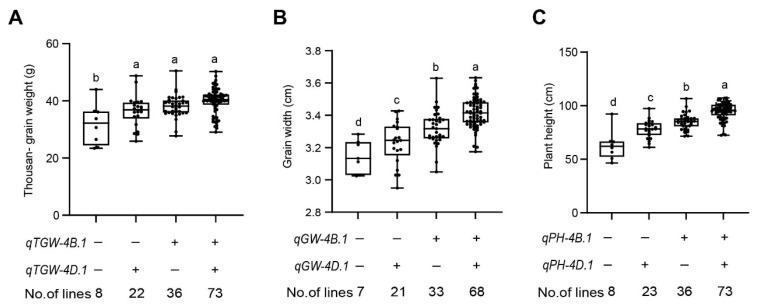
Additive effects of QTL on TGW, GW, and PH in the ZK331 × ND399 RIL population. Analyses were performed using the BLUE datasets. (**A**–**C**) Additive effects on TGW (**A**), GW (**B**), and PH (**C**). The presence (+) and absence (−) of favorable alleles were determined based on genotypes of flanking markers for each QTL. Different letters indicate statistically significant differences [analysis of variance (ANOVA), the least significant difference (LSD) method].

We evaluated the phenotypic traits of wheat plants with varying combinations of QTL, *qTGW*/*GW*/*PH-4B.1* and *qTGW*/*GW*/*PH-4D.1*, on chromosomes 4B and 4D, controlling TGW, GW, and PH. The absence of both loci resulted in the lowest TGW (31.8 g), GW (3.1 mm), and PH (58.8 cm). In contrast, the presence of the locus *qTGW*/*GW*/*PH-4D.1* alone led to a significant increase in these traits, with TGW of 36.4 g, GW of 3.2 mm, and PH reaching 78.0 cm. Similarly, the locus *qTGW*/*GW/PH-4B.1* alone conferred a TGW of 38.1 g, GW of 3.3 mm, and PH of 85.1 cm. The combination of both loci yielded the highest values across all traits, with a TGW of 40.0 g, GW of 3.4 mm, and PH of 95.1 cm. (Figure 4A–C and Figure S4). These results indicate the additive and synergistic effects of these QTL on the enhancement of wheat yield-related traits.

### 2.7. Analysis of the Annotated Genes in the Target Genomic Interval of qTGW/GW/PH-4B.1 and qTGW/GW/PH-4D.1

Based on the positions of two flanking markers, the chromosome interval of locus *qTGW/GW/PH-4B.1* was mapped to between 28,739,948 and 32,174,878 bp in the Chinese Spring reference genome sequences v1.1 (IWGSC, accessed on 14 March 2025 http://www.wheatgenome.org/). A total of 21 high-confidence genes were identified in this interval (Table S4). Among these, two genes, *TraesCS4B02G042700* and *TraesCS4B02G043100*, were predicted to influence plant height and yield. *TraesCS4B02G042700* encodes a transcription factor (T1, a regulator of plant growth), while *TraesCS4B02G043100* (*Rht-B1*) encodes a DELLA protein (a key regulator of gibberellin signaling involved in dwarfing). Expression analysis across different tissues revealed that *TraesCS4B02G043100* was highly expressed in stems and grains (Figure S5), suggesting it is a potential candidate gene.

In the *qTGW/GW/PH-4D.1* interval, 105 high-confidence genes were identified, of which six may be related to plant height and yield (Table S5). *TraesCS4D02G030900* and *TraesCS4D02G033100* are associated with activity and signal transduction of auxin. *TraesCS4D02G033800* is involved in cytokinin metabolism. *TraesCS4D02G036000* regulates ethylene signal transduction. *TraesCS4D02G040100* is a transcription factor regulating plant architecture. *TraesCS4D02G040400* is the *Rht-D1* gene encoding a DELLA protein. Expression analysis of these genes showed that *TraesCS4D02G040400* was highly expressed in stems and seeds (Figure S6), indicating it as the potential candidate gene.

## 3. Discussion

The identification of genetic loci that control grain traits and plant height, which are key agronomic traits influencing yield potential, is essential for developing high-yielding wheat varieties [34]. This study identified several QTL that significantly affect TGW, GL, GW, and PH on various wheat chromosomes. For TGW, the main chromosomes were 1B, 2B, 3B, 4B, and 4D; for GL, 1B, 2B, 3A, 3B, 4A, and 7A; for GW, 3D, 4B, and 4D; and for PH, 1A, 3D, 4B, 4D, 5A, and 7A. These results provide valuable insights into the genetic basis of these traits and offer a foundation for their targeted improvement in wheat breeding programs.

Our results reveal that *qGL-1B.1* on chromosome 1B overlaps with previously identified loci, such as *QKl.sdau-1B.2*, indicating a conserved genomic region associated with GL [29]. This conservation suggests that specific genomic regions consistently influence GL across diverse studies and wheat populations. Additionally, we identified a novel QTL, *qGL-7A.1* for GL, on chromosome 7A near the 607.58–617.67 Mb interval. This QTL differs from a previously identified grain length QTL on 7A, *qKL-7A.1*, located within the 303.63–379.43 Mb interval [35]. This distinction indicates that *qGL-7A.1* is likely a newly discovered locus that expands our understanding of the genetic control of grain length in wheat.

*qTGW-4B.1* for TGW was mapped to a genomic region on chromosome 4B that overlaps with a previously reported QTL, *QTGW.baafs-4B* [25]. The QTL site *QTgw.caas-4B* (22.92–32.17 Mb) on 1000-grain weight was also reported in previous articles [26], which coincided with the locus located in this study. Additionally, two novel QTL, *qTGW-2B.1* and *qTGW-3B.1*, were consistently detected across diverse environments. *qTGW-4B.1*, *qGW-4B.1*, and *qPH-4B.1* were co-located in the 28.73–32.17 Mb interval on chromosome 4B. A similar pattern was observed on chromosome 4D, where *qTGW-4D.1*, *qGW-4D.1*, and *qPH-4D.1* were located within the 13.21–48.70 Mb interval. These results suggest pleiotropic effects of these loci, simultaneously influencing TGW, GW, and PH. This co-location provides opportunities for the concurrent improvement of these traits in breeding programs. The identification of several novel QTL, such as *qTgw-2B.1* and *qTgw-3B.1*, demonstrated consistent expression across multiple environments, further indicating the potential of these novel loci for enhancing yield stability in wheat.

Loci *qPH-4B.1* and *qPH-4D.1* for plant height were mapped to regions corresponding to the well-known “Green Revolution” genes *Rht1-B1* and *Rht1-D1*, respectively [36]. These genes have long been used in modern wheat breeding to reduce plant height and increase yield potential. Additionally, *qPH-5A.1* was identified as a distinct QTL, separate from the previously reported *QHt.fcu-5A* located on chromosome 5AL [37]. This suggests that *qPH-5A.1* is a novel locus for plant height, offering new possibilities for the genetic improvement of this trait.

The ideal plant height of winter wheat is an important index affecting yield, lodging resistance, and environmental adaptability [36,38]. The ideal plant height for winter wheat, usually between 70 cm and 80 cm, can reduce the risk of lodging and improve the efficiency of water and nutrient use while ensuring sufficient photosynthetic area and thus maintaining relatively high biomass [39,40]. We found that when only the locus *qPH-4B.1* or *qPH-4D.1* was present in the combined genotype, the plant height was 85.18 cm and 78.02 cm, respectively, which was more suitable for field planting.

## 4. Materials and Methods

### 4.1. Plant Materials

ZK331 is an advanced breeding line recognized for its high yield potential and resistance to major wheat diseases, while ND399 exhibits strong lodging resistance, effective grain filling, and strong tillering ability. To combine these desirable traits, a cross was made between ZK311 and ND399, producing an F_2:6_ RIL population consisting of 146 lines using the single seed descent method. This population was used to identify and characterize QTL associated with key agronomic traits.

### 4.2. Field Experiments and Trait Phenotyping

Field trials were conducted across six environments with diverse climatic and soil conditions. These environments included Shijiazhuang, Hebei Province (114°29′ E, 38°03′ N) in 2020 and 2021 (environments E1 and E2); Qingshui, Gansu Province (104°35′ E, 34°05′ N) in 2021 (E3); Anyang, Henan Province (114°20′ E, 36°00′ N) in 2021 (E4); Chengdu, Sichuan Province (104°03′ E, 30°39′ N) in 2021 (E5); and Hangzhou, Zhejiang Province (120°12′ E, 30°14′ N) in 2021 (E6). A randomized complete block design with three replicates per line was used in all trials to reduce experimental variability. Planting schedules were adapted to local practices. Standard agricultural practices, including weed control, disease and pest management, fertilization, and irrigation, were applied in accordance with local farming customs.

At the grain-filling stage, PH was measured from the ground to the tip of spikes (excluding awns) on three plants per replicate. Upon maturity, a representative sample of 10 plants from each replicate was collected to determine TGW by weighing three sets of 100 grains from each replicate. Grain dimensions (GL and GW) were measured by scanning 50 grains using a seed-determining instrument (Zhejiang Top Instrument Co., Ltd., Hangzhou, China). The mean values for each trait across replicates were calculated for data analysis and QTL mapping.

### 4.3. Genetic Map Construction and QTL Analysis

Genomic DNA was extracted from young leaves of ZK331, ND399, and their progeny RILs using the cetyltrimethylammonium bromide (CTAB) method [41]. Genotyping was performed using the 16 K GenoBaits Wheat SNP Array (MolBreeding, Shijiazhuang, China). Raw sequencing reads were processed using fastp v0.20.0 [42], then aligned to the Chinese Spring (CS) reference genome (RefSeq v1.0) using the Burrows–Wheeler Aligner (BWA). High-quality SNPs were filtered using GATK v3.5 [43,44], excluding variants with a read depth below 5. SNP loci with minor allele frequencies between 0.2 and 0.8 were categorized as heterozygous, while all others were considered homozygous.

A genetic linkage map was constructed using IciMapping 4.2, with one marker per bin selected for map construction [45]. The inclusive composite interval mapping (ICIM) method implemented in IciMapping 4.2 was used to identify QTL. The LOD threshold was set at 2.5, chosen to balance statistical significance and practical detection power, minimizing false positives while effectively identifying true QTL. This threshold was determined based on 1000 permutations to control the genome-wide false discovery rate at 5% (*p* < 0.05).

### 4.4. Statistical Analysis

Statistical analyses were conducted for traits including TGW, GL, GW, and PH. Average values were calculated using Microsoft Excel. For combined QTL detection, correlation, and normal distribution analyses, BLUEs were obtained using the ANOVA function in QTL IciMapping 4.2 [45]. Broad-sense heritability (*h*^2^) was calculated using R 4.4.1 software with standard deviations and correlation coefficients for each trait. Heritability was estimated using the formula *h*^2^ *=* VG/VP, where VG is genetic variance and VP is phenotypic variance.

## 5. Conclusions

We identified multiple QTL associated with key agronomic traits, i.e., TGW, GL, GW, and PH, in the ZK331 × ND399 RIL population. The genetic loci were mapped across various chromosomes, with significant QTL detected on chromosomes 1B, 2B, 3B, 4B, and 4D for TGW; 1B, 2B, 3A, 3B, 4A, and 7A for GL; 3D, 4B, and 4D for GW; and 1A, 3D, 4B, 4D, 5A, and 7A for PH. Several QTL, such as *qGL-7A.1* and *qTGW-2B.1*, were novel and consistently expressed across environments, indicating their potential for enhancing yield stability. The co-localization of QTL for different traits, particularly on chromosomes 4B and 4D, suggests pleiotropic effects that could be utilized for concurrent trait improvement in breeding programs. These results provide a robust genetic framework for targeted wheat breeding, aiming to optimize yield and lodging resistance while ensuring environmental adaptability.

## Figures and Tables

**Table 1:** Phenotypic variation in thousand-grain weight (TGW), grain length (GL), grain width (GW), and plant height (PH) in Zhongke 331, Nongda 399, and their derived recombinant inbred lines (RILs) under various environmental conditions.

		ZK331	ND399	Min.	Max.	Average	SD	Skewness	Kurtosis	*h* ^2^
TGW (g)	E1	53.4	45.6	36.5	57.1	46.9	3.60	−0.11	0.16	77.5
	E2	52.8	46.3	37.7	63.0	49.9	4.58	0.04	−0.09	
	E3	37.2	35.4	18.0	45.3	35.3	4.82	−0.54	0.78	
	E4	36.5	34.2	21.0	51.0	39.6	5.41	−0.36	0.32	
	E5	43.7	41.2	22.9	54.7	41.3	5.66	0.14	0.22	
	E6	37.8	34.2	9.7	53.7	28.8	7.95	0.32	0.26	
GL (mm)	E1	6.7	6.3	6.0	7.6	6.8	0.31	0.21	0.18	93.2
	E2	7.1	6.7	6.2	7.9	6.9	0.29	0.55	0.87	
	E3	6.8	6.2	6.1	7.5	6.6	0.29	−0.66	0.39	
	E4	6.8	6.2	6.1	7.9	6.8	0.30	0.67	1.18	
	E5	6.9	6.2	5.7	7.3	6.4	0.30	0.48	0.49	
	E6	6.8	6.3	5.5	7.4	6.4	0.39	0.31	−0.15	
GW (mm)	E1	3.3	2.8	3.0	3.8	3.5	0.14	−0.61	1.1	75.9
	E2	3.7	3.2	3.1	4.0	3.6	0.14	−0.58	1.92	
	E3	3.1	2.8	2.6	3.8	3.2	0.20	−0.15	0.48	
	E4	3.6	3.2	2.9	3.8	3.3	0.17	−0.06	0.09	
	E5	3.6	3.2	2.8	3.9	3.4	0.18	−0.08	0.4	
	E6	3.4	3.0	2.3	4.0	3.0	0.29	0.25	0.74	
PH (cm)	E2	72.0	68.0	40.3	99.7	74.3	12.57	−0.61	0.47	90.5
	E3	71.7	67.8	41.7	103.3	83.1	11.87	−0.86	0.58	
	E4	76.5	71.2	47.0	129.3	96.7	17.23	−0.66	0.54	
	E5	78.2	73.6	54.0	120.7	92.7	10.95	−0.55	1.18	
	E6	68.3	64.9	50.0	120.3	93.8	13.98	−0.4	−0.07	

E1 and E2 represent Shijiazhuang, Hebei Province (2020 and 2021); E3 represents Qingshui, Gansu Province (2021); E4 represents Anyang, Henan Province (2021); E5 represents Chengdu, Sichuan Province (2021); and E6 represents Hangzhou, Zhejiang Province (2021).

**Table 2:** Stable QTL for thousand-grain weight (TGW), grain length (GL), grain width (GW), and plant height (PH) identified from different environments in the Zhongke 331 × Nongda 399 RIL population.

Trait	QTL	Environment	Position	Left Marker	Right Marker	LOD	PVE ^a^	Add ^b^	Previous Study
TGW	*qTGW-1B.1*	E5	82	*1B_637885015*	*1B_641922827*	3.65	11.38	1.90	
		E6	85	*1B_637885015*	*1B_641922827*	4.40	14.44	2.98	
	*qTGW-2B.1*	E3	63	*2B_39795004*	*2B_67981606*	4.91	14.63	1.87	
		E5	64	*2B_67981922*	*2B_68424868*	5.01	11.20	2.12	
	*qTGW-3B.1*	E3	59	*3B_44543422*	*3B_50191187*	3.81	11.86	1.71	*QTgw.cib-3B.1* [24]
		E5	57	*3B_43599467*	*3B_44857581*	3.18	10.99	1.85	
	*qTGW-4B.1*	E4	55	*4B_28739948*	*4B_32174878*	5.94	18.65	2.46	*QTGW.baafs-4B* [25]; *QTgw.caau-4B* [26]
		E6	54	*4B_28739948*	*4B_32174878*	4.62	14.41	3.34	*QTgw.cau-4B.2* [27]; *QTgw.cau-4B-1* [28]
	*qTGW-4D.1*	E1	40	*4D_19090268*	*4D_48697668*	2.64	8.82	−1.42	
		E2	37	*4D_13215376*	*4D_19090268*	2.75	7.93	−1.73	
GL	*qGL-1B.1*	E1	82	*1B_637885015*	*1B_641922827*	4.86	14.47	0.12	*QKl.sdau-1B.2* [29]
		E2	80	*1B_564852828*	*1B_637584804*	7.15	21.14	0.13	
		E3	83	*1B_637885015*	*1B_641922827*	8.54	24.27	0.15	
		E4	80	*1B_564852828*	*1B_637584804*	5.75	20.07	0.13	
		E5	83	*1B_637885015*	*1B_641922827*	6.81	19.39	0.14	
	*qGL-2B.1*	E3	59	*2B_39795004*	*2B_67981606*	3.49	8.89	0.11	
		E6	63	*2B_39795004*	*2B_67981606*	4.78	8.69	0.15	
	*qGL-2B.2*	E2	161	*2B_690211230*	*2B_694440260*	2.67	10.20	−0.09	*QGl.cau-2B.2* [30]
		E5	161	*2B_690211230*	*2B_694440260*	3.22	11.56	−0.10	
	*qGL-2D.1*	E3	107	*2D_531701660*	*2D_562710587*	3.29	10.96	0.11	*QKL.sicau-SSY-2D* [31]
		E4	104	*2D_531701660*	*2D_562710587*	2.93	10.96	0.11	
	*qGL-3A.1*	E2	258	*3A_741822204*	*3A_746542290*	3.11	9.70	0.10	
		E3	268	*3A_746542290*	*3A_747278706*	3.04	8.37	0.09	
		E4	268	*3A_746542290*	*3A_747278706*	2.74	8.73	0.09	
	*qGL-3B.1*	E3	45	*3B_31983800*	*3B_34669808*	5.22	15.86	0.12	
		E4	44	*3B_31983800*	*3B_34669808*	3.21	9.71	0.09	
	*qGL-3B.2*	E3	188	*3B_757269550*	*3B_759449813*	2.84	7.74	0.08	
		E6	188	*3B_757269550*	*3B_759449813*	19.92	11.10	0.35	
	*qGL-4A.1*	E2	50	*4A_466204879*	*4A_468921463*	3.05	11.66	0.09	
		E4	50	*4A_466204879*	*4A_468921463*	4.14	13.38	0.11	
	*qGL-7A.1*	E3	148	*7A_607579794*	*7A_617666459*	12.10	17.27	0.12	
		E6	148	*7A_607579794*	*7A_617666459*	8.73	16.25	0.17	
GW	*qGW-3D.1*	E2	144	*3D_538481562*	*3D_551980404*	3.23	10.03	0.04	
		E4	142	*3D_538481562*	*3D_551980404*	3.78	11.34	0.06	
	*qGW-4B.1*	E1	55	*4B_28739948*	*4B_32174878*	2.75	8.45	0.04	*QGw.caau-4B* [26]
		E2	51	*4B_24900757*	*4B_28739948*	4.07	13.67	0.05	
		E3	45	*4B_24900757*	*4B_28739948*	4.07	13.56	0.08	
		E4	47	*4B_24900757*	*4B_28739948*	3.54	11.63	0.07	
		E6	47	*4B_24900757*	*4B_28739948*	3.62	12.59	0.11	
	*qGW-4D.1*	E5	36	*4D_13215376*	*4D_19090268*	3.18	9.41	-0.07	
		E6	45	*4D_19090268*	*4D_48697668*	3.08	9.98	−0.13	
PH	*qPH-1A.1*	E4	25	*1A_6791455*	*1A_14475390*	2.93	9.15	4.95	
		E5	27	*1A_14475390*	*1A_23131762*	2.65	9.36	3.17	
	*qPH-3D.1*	E5	143	*3D_538481562*	*3D_551980404*	3.19	9.29	3.61	
		E6	143	*3D_538481562*	*3D_551980404*	5.41	15.70	5.00	
	*qPH-4B.1*	E2	53	*4B_28739948*	*4B_32174878*	5.67	16.81	4.73	*QPH.baafs-4B* [25]
		E3	53	*4B_28739948*	*4B_32174878*	5.82	17.60	7.52	*QPh.cau-4B.2* [27]
		E4	53	*4B_28739948*	*4B_32174878*	6.02	17.25	4.85	*QPh-4B1* [32]
		E5	54	*4B_28739948*	*4B_32174878*	9.38	26.24	8.32	*QPH-caas-4BS.2* [33]
		E6	53	*4B_28739948*	*4B_32174878*	15.81	21.48	6.99	
	*qPH-4D.1*	E2	40	*4D_19090268*	*4D_48697668*	11.37	29.91	−8.07	*QTgw.cau-4D.1* [27]
		E3	37	*4D_13215376*	*4D_19090268*	16.42	40.51	−9.31	*QPh-4D* [32]
		E4	37	*4D_13215376*	*4D_48697668*	13.41	39.36	−11.53	*QPH.caas-4DS.1* [33]
		E5	39	*4D_19090268*	*4D_48697668*	12.22	34.29	−7.49	
		E6	40	*4D_19090268*	*4D_48697668*	10.79	31.43	−9.35	
	*qPH-5A.1*	E2	74	*5A_532377854*	*5A_535830656*	4.18	12.78	3.88	
		E4	74	*5A_532377854*	*5A_535830656*	5.01	15.12	5.88	
		E5	75	*5A_535032560*	*5A_539540555*	4.10	11.87	3.90	
		E6	75	*5A_535032560*	*5A_539540555*	4.17	12.23	5.04	
	*qPH-7A.1*	E2	208	*7A_701630471*	*7A_724015398*	5.11	6.82	2.90	
		E4	208	*7A_701630471*	*7A_724015398*	2.60	3.94	3.74	
		E6	208	*7A_701630471*	*7A_724015398*	3.52	4.00	2.80	

^a^ Phenotypic variance effect (PVE) explained by one QTL. ^b^ Positive values indicate that alleles from Zhongke 331 increased the trait values, while the negative values indicate that the alleles from Nongda 399 increased the corresponding trait values.

## Data Availability

Data are contained within the article and Supplementary Materials.

## Supplementary Materials

The following supporting information can be downloaded at https://www.mdpi.com/article/10.3390/ijms26083526/s1.
