# Supplementary figures and images for: Identification of QTL for Grain Traits and Plant Height Using the Recombinant Inbred Line Population Derived from the Cross of Zhongke 331 × Nongda 399
Source: Int J Mol Sci. 2025 Apr 9;26(8):3526. doi: 10.3390/ijms26083526 (PMC12027352; doi:10.3390/ijms26083526)

## Supplementary Figures

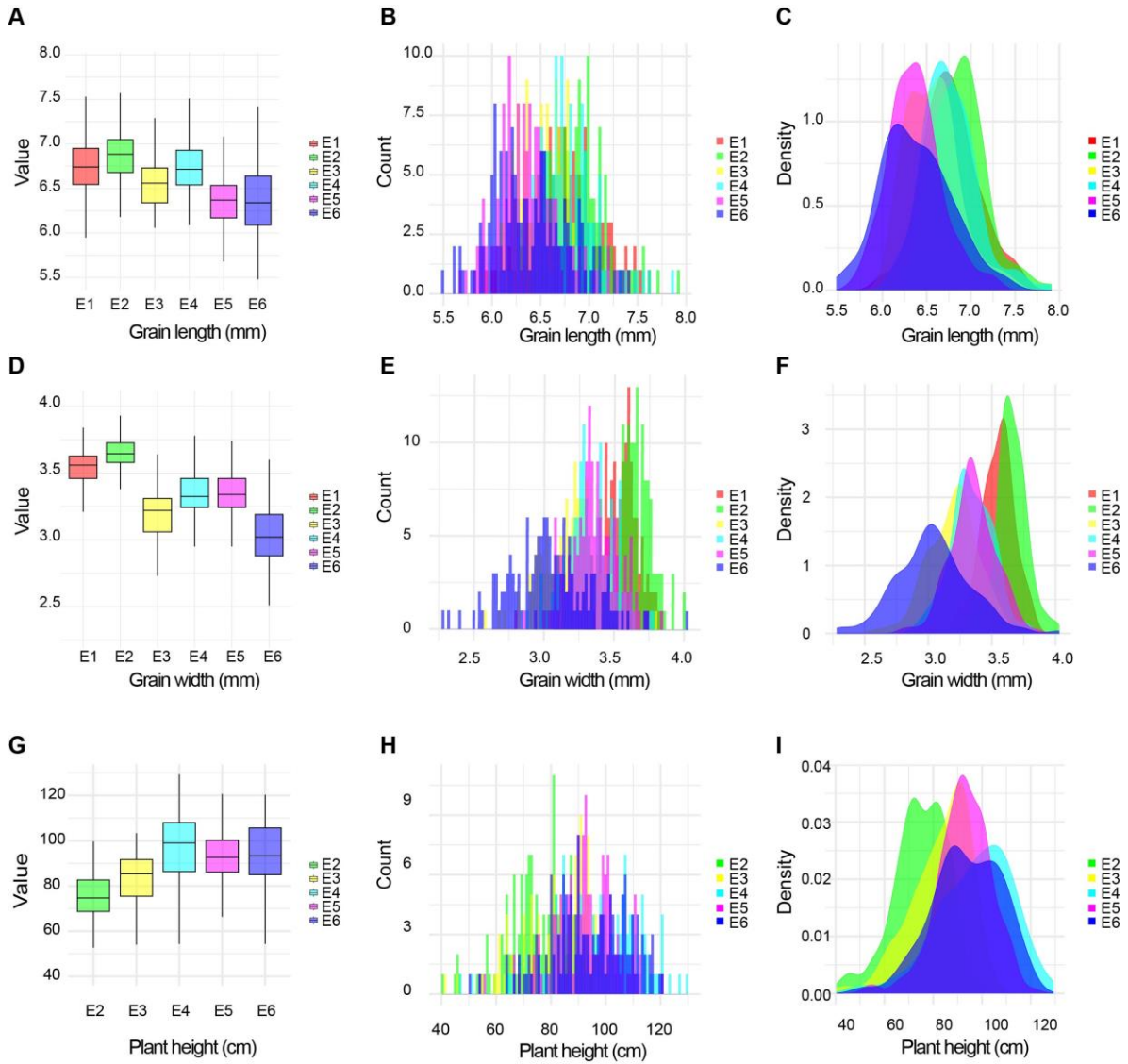

Figure S1

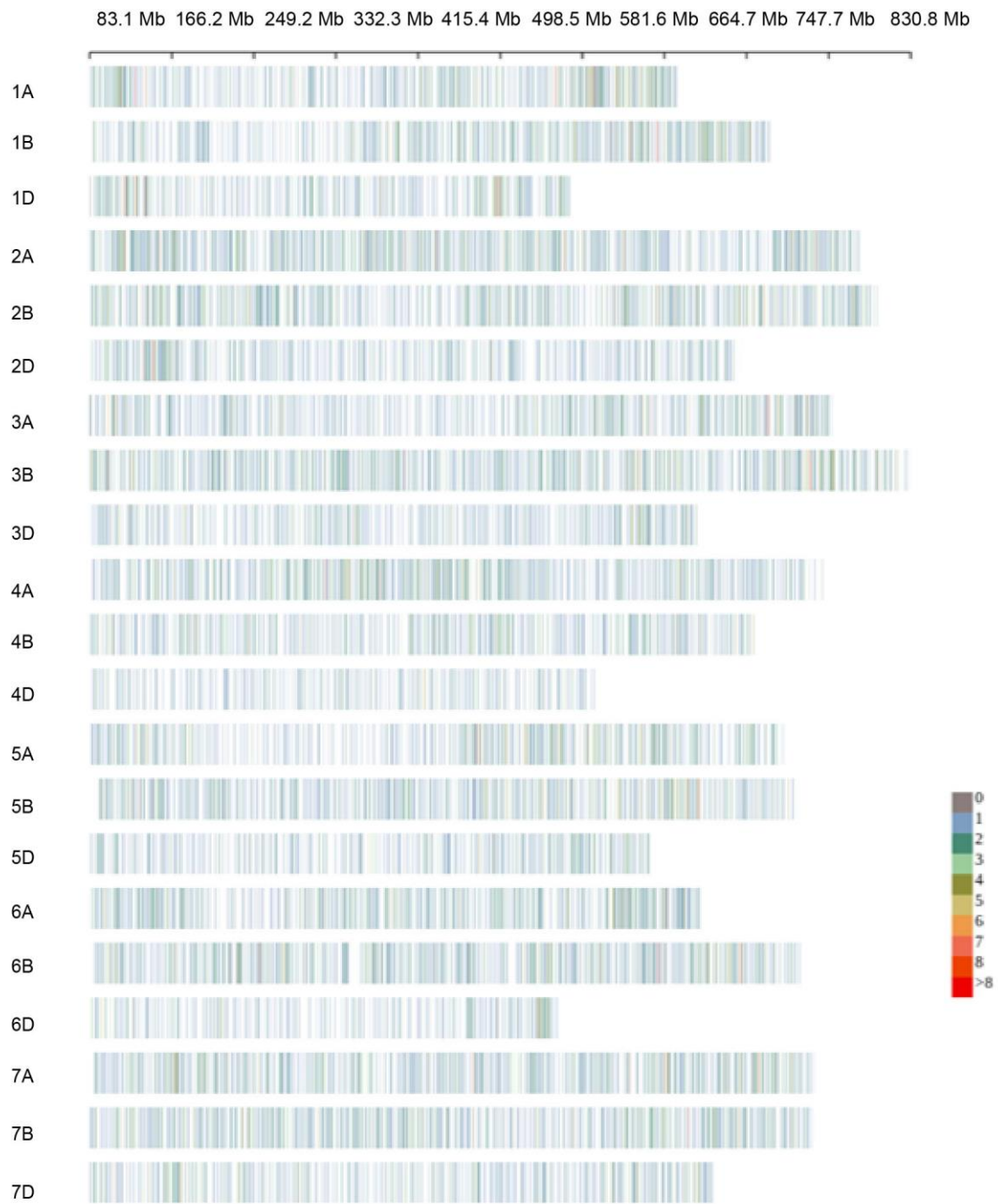

**Figure S2**

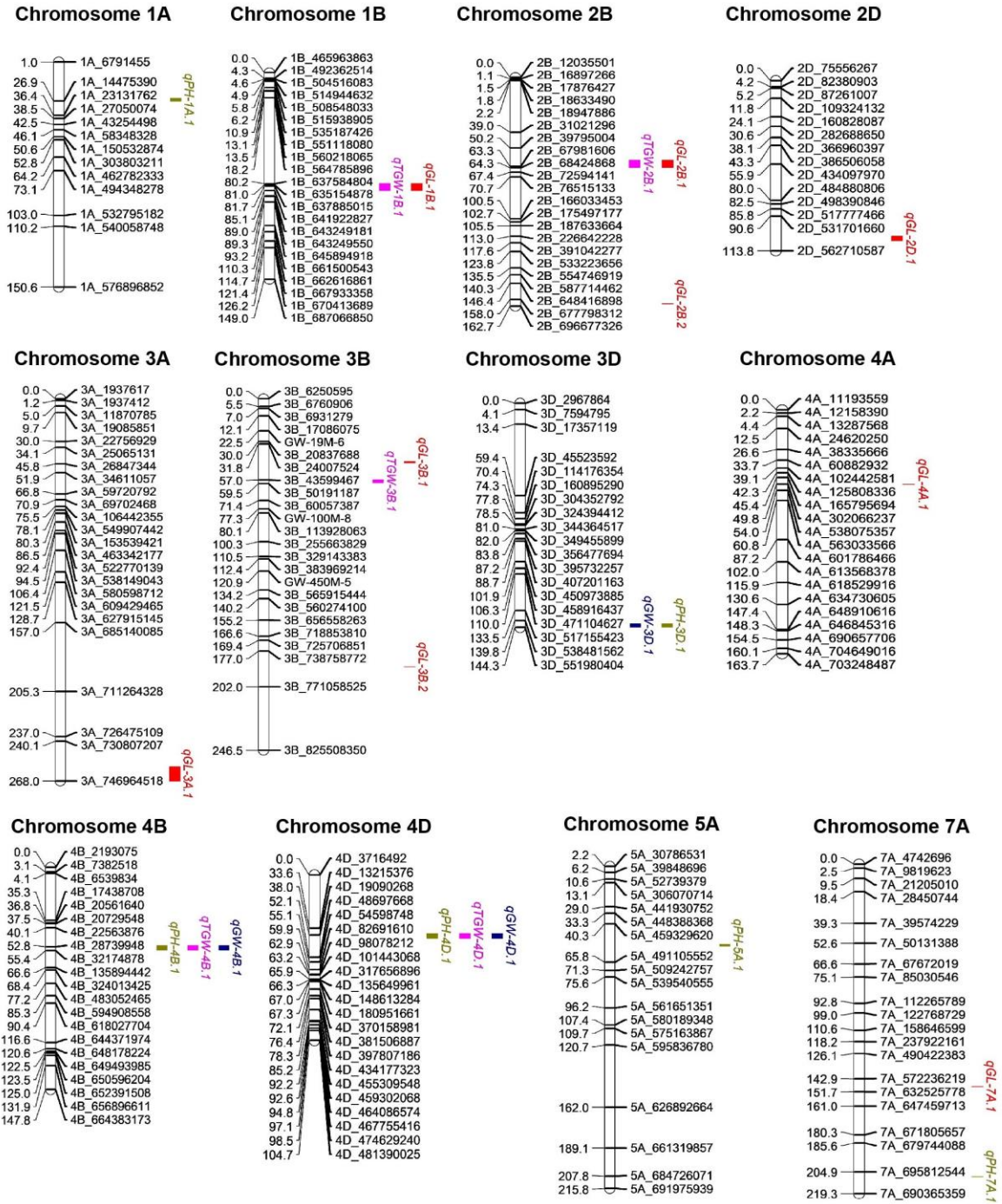

Figure S3

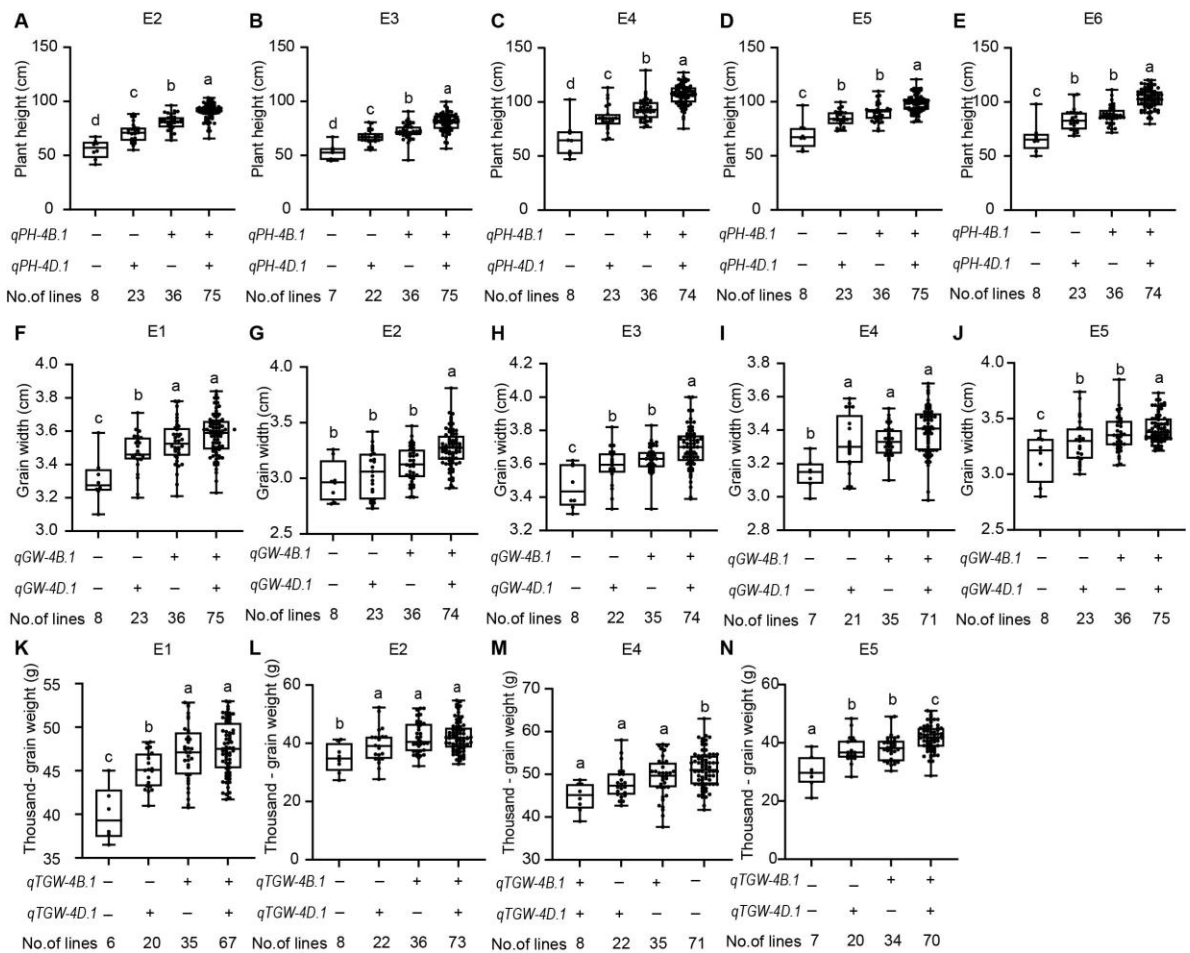

Figure S4

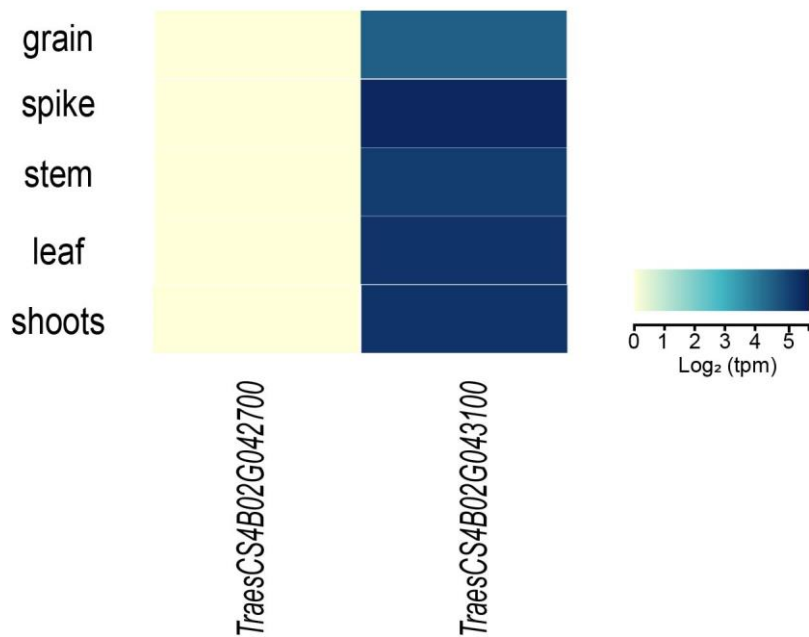

Figure S5

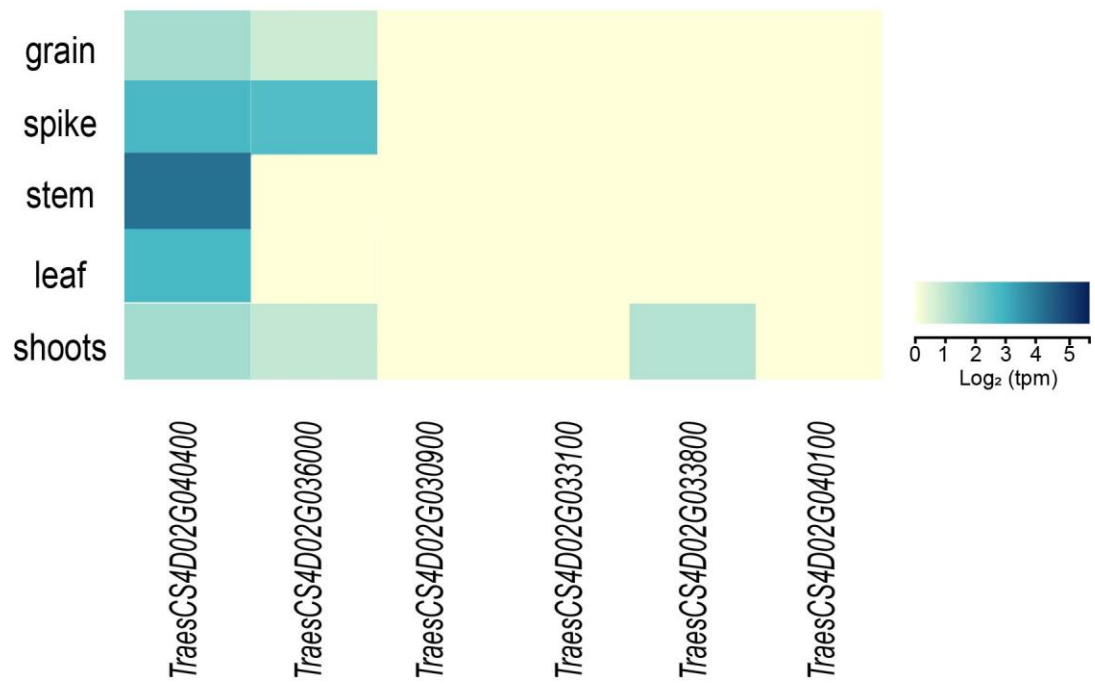

Figure S6

Supplement: Supplementary file 1 [file ijms-26-03526-s001.zip › ijms-3510237-Supplementary Figures.pdf]
